# Biomarkers and subtypes of cancer

**DOI:** 10.18632/aging.100741

**Published:** 2015-05-03

**Authors:** Maud H.W. Starmans, Paul C. Boutros

**Affiliations:** Informatics & Biocomputing Program, Ontario Institute for Cancer Research, Toronto, Canada

The identification of prognostic and predictive biomarkers is a key research area in medicine. These biomarkers aim to contribute to personalize medicine. Ultimately, in personalized medicine treatment will be tailored towards each patient's specific disease and genetics to optimize treatment outcome and minimize side effects. In cancer research large efforts are made to screen for biological entities like gene mutations and transcription-based biomarkers for this purpose, however the identified markers are most of the time not accurate enough for clinical use. Recently we have shown that confounding factors play an important role in the limited performance of such (bio)markers [1]. Mutations in the RAS gene, a gene frequently mutated in lung cancer, were not prognostic [2], however they largely influenced accuracy of transcription-based biomarkers for non-small cell lung cancer. Taking RAS mutations to define patient subgroups and define transcription-based biomarkers for these specific patient subgroups resulted in an increase in prognostic power. While screening for prognostic or predictive markers it will thus be key to be aware of and correct for potential confounders. Therefore to create clinically useful biomarkers it will be detrimental to define clinically relevant patient subgroups rather than generalize across patients.

This general principle might apply to a broad range of other variables and studies. For example, one can imagine different biomarkers being optimal in older *vs*. younger patients, in men *vs*. women and especially based on a broad range of other tumour genetic information. To this last point, large studies such as those initiated by The Cancer Genome Atlas (TCGA) and the International Cancer Genome Consortium (ICGC) will provide a wealth of data to exploit these findings. These studies can be used to define clinically-relevant patient subgroups based on genetic heterogeneity, rather than investigating single entities. For example, one can imagine systematic studies to identify genes that, while not themselves prognostic, confound the accuracy of other prognostic markers. Or, indeed, confound the accuracy of other biomarkers entirely: diagnostic or predictive markers, or markers for monitoring disease progress could all follow this general template.

To perform such analyses, it will be critical to rigorously assess the information content of different classes of biomarkers in different clinical situations. For example, we established interplay between RAS mutation and expression of a set of 14 genes; a gene expression-based classifier could be used to predict RAS mutation status. A large number of random gene sets were used to show this RAS predictor had optimal performance. Further large permutation studies, testing millions of random gene sets for their prognostic power, established that predicting prognosis for patients with RAS mutations should be done with different gene sets than for patients without RAS mutations. Testing large sets of random gene sets also provides valuable information for performance of transcriptome-based biomarkers. Comparing performance of the biomarker against the performance distribution of the random gene sets will immediately show whether these perform better than random and are worthwhile proceeding with [3, 4].

Taken together, these data point at a sea-change in the development of biomarkers. Rather than simply focusing on finding the best “signature” to predict a specific clinical event [5, 6], we will look to further sub-stratify patient populations into subtypes that can be accurately prognosed. Indeed, while these subtypes themselves may not be inherently informative, they may provide the structure or framework upon which more accurate biomarkers can be developed. We can foresee the adoption of information content methods like those described above to try to identify proactively specific genomic events that mark groups of patients with coherently predictable clinical outcome.
